# Incorporation of a machine learning pathological diagnosis algorithm into the thyroid ultrasound imaging data improves the diagnosis risk of malignant thyroid nodules

**DOI:** 10.3389/fonc.2022.968784

**Published:** 2022-12-08

**Authors:** Wanying Li, Tao Hong, Jianqiang Fang, Wencai Liu, Yuwen Liu, Cunyu He, Xinxin Li, Chan Xu, Bing Wang, Yuanyuan Chen, Chenyu Sun, Wenle Li, Wei Kang, Chengliang Yin

**Affiliations:** ^1^ Center for Management and Follow-up of Chronic Diseases, Xianyang Central Hospital, Xianyang, China; ^2^ Pediatric Surgery Ward, Fuwai Hospital Chinese Academy of Medical Sciences, Shenzhen, China; ^3^ Ultrasound Interventional Department, Xianyang Central Hospital, Xianyang, China; ^4^ Clinical Medical Research Center, Xianyang Central Hospital, Xianyang, China; ^5^ Department of Orthopaedic Surgery, The First Affiliated Hospital of Nanchang University, Nanchang, China; ^6^ Department of Chronic Disease and Endemic Disease Control Branch, Xiamen Municipal Center for Disease Control and Prevention, Xiamen, China; ^7^ School of Statistics, RENMIN University of China, Beijing, China; ^8^ AMITA Health Saint Joseph Hospital Chicago, Chicago, IL, United States; ^9^ State Key Laboratory of Molecular Vaccinology and Molecular Diagnostics and Center for Molecular Imaging and Translational Medicine, School of Public Health, Xiamen University, Xiamen, China; ^10^ Department of Mathematics, Physics and Interdisciplinary Studies, Guangzhou Laboratory, Guangzhou, Guangdong, China; ^11^ Faculty of Medicine, Macau University of Science and Technology, Macao, Macao SAR, China

**Keywords:** thyroid nodules, malignant, machine learning, predictive model, web calculator

## Abstract

**Objective:**

This study aimed at establishing a new model to predict malignant thyroid nodules using machine learning algorithms.

**Methods:**

A retrospective study was performed on 274 patients with thyroid nodules who underwent fine-needle aspiration (FNA) cytology or surgery from October 2018 to 2020 in Xianyang Central Hospital. The least absolute shrinkage and selection operator (lasso) regression analysis and logistic analysis were applied to screen and identified variables. Six machine learning algorithms, including Decision Tree (DT), Extreme Gradient Boosting (XGBoost), Gradient Boosting Machine (GBM), Naive Bayes Classifier (NBC), Random Forest (RF), and Logistic Regression (LR), were employed and compared in constructing the predictive model, coupled with preoperative clinical characteristics and ultrasound features. Internal validation was performed by using 10-fold cross-validation. The performance of the model was measured by the area under the receiver operating characteristic curve (AUC), accuracy, precision, recall, F1 score, Shapley additive explanations (SHAP) plot, feature importance, and correlation of features. The best cutoff value for risk stratification was identified by probability density function (PDF) and clinical utility curve (CUC).

**Results:**

The malignant rate of thyroid nodules in the study cohort was 53.2%. The predictive models are constructed by age, margin, shape, echogenic foci, echogenicity, and lymph nodes. The XGBoost model was significantly superior to any one of the machine learning models, with an AUC value of 0.829. According to the PDF and CUC, we recommended that 51% probability be used as a threshold for determining the risk stratification of malignant nodules, where about 85.6% of patients with malignant nodules could be detected. Meanwhile, approximately 89.8% of unnecessary biopsy procedures would be saved. Finally, an online web risk calculator has been built to estimate the personal likelihood of malignant thyroid nodules based on the best-performing ML-ed model of XGBoost.

**Conclusions:**

Combining clinical characteristics and features of ultrasound images, ML algorithms can achieve reliable prediction of malignant thyroid nodules. The online web risk calculator based on the XGBoost model can easily identify in real-time the probability of malignant thyroid nodules, which can assist clinicians to formulate individualized management strategies for patients.

## Introduction

The incidence of sonographically detected thyroid nodules is increasing in individuals; approximately 50% to 68% can be detected in healthy individuals. Most of these nodules are benign and asymptomatic (1–3), and only about 8% to 16% are malignant nodules (4–6). Due to the complexity and diversity of thyroid nodules, it is challenging for doctors to distinguish which nodules harbor clinically relevant malignancies (7). For more than 30 years, ultrasound and fine-needle aspiration (FNA) cytology were the traditional diagnostic methods as the cornerstones in the clinical management of patients with thyroid nodules (8).

FNA provides the most effective and practical diagnostic information for evaluating whether a nodule is malignant to reach a definitive diagnosis, which has traditionally been used to meet this purpose (9, 10). However, approximately 50% of all biopsied nodules proved to be benign and grew indolent with non-aggressive behavior (6, 11, 12). Moreover, biopsies in one out of seven thyroid nodules may not yield final cytological results and usually require repeated biopsies or additional evaluation (13). Obviously, it is not cost-effective to submit all these lesions to FNA.

As a non-invasive, low-cost, and convenient technique for thyroid nodule detection, ultrasound is widely accepted as the preferred imaging method for the diagnosis and monitoring of thyroid nodules. Therefore, ultrasonography has been considered as having a greater role in determining the need for FNA and follow-up planning (2, 7). In order to improve the accuracy of ultrasound-based diagnosis, various available ultrasound-based risk stratification systems have already been proposed by many national and international thyroid associations, such as the ACR TIRADS, the French TIRADS, the Korea-TIRADS, and the EU-TIRADS (14). The most commonly used thyroid nodule classification system is the Thyroid Imaging Reporting and Data System (TIRADS) developed by the American College of Radiology (ACR). However, the limitations of these systems include that subjective assessment of nodules (15) is inferior to the personal judgment by experts (8) and that different classification systems for the same thyroid nodules may yield varying results (16), which cannot be ignored. There is an urgent need to develop an improved and reliable diagnostic method to distinguish benign and malignant thyroid nodules, which could help reduce the number of unnecessary biopsies or diagnostic surgery without jeopardizing the detection of clinically relevant malignant thyroid nodules.

A predictive model based on machine learning (ML) algorithms, designed to “learn” from clinical and sonographic datasets and predict the nature of thyroid nodules, is in some cases more robust than human experts (17), and as a result, ML algorithms have been widely used to classify thyroid nodules objectively (18–20). However, previous studies have classified thyroid nodules by analyzing thyroid ultrasound images. The purpose of the present study was to develop ML-ed models for predicting malignant lumps based on the database of clinical characteristics and ultrasound features of thyroid nodules confirmed by pathological examination in Chinese populations. Compared with using only image analysis, our ML-ed predictive models not only integrated ultrasound features but also included clinical features of patients with thyroid nodules, which may be more comprehensive and convenient, especially for clinicians and patients. It can carry out individualized treatment and management based on the received ultrasound reports. The new model obtained could be used to predict the malignant risk of thyroid nodules in individuals online *via* a web calculator.

## Materials and methods

The retrospective study followed the tenets of the Declaration of Helsinki and was approved by the Ethics Committee of Xianyang Central Hospital (No. 2022-IRB-68). All the study participants provided written informed consent, which waived the requirement for informed patient consent because data for all subjects were anonymized.

### Collection of patients

A total of 9,999 consecutive patients with thyroid nodules who underwent FNA cytology or surgical procedure at Xianyang Central Hospital from the year 2000 to 2020 were included in our study. All included participants met the following inclusion criteria: 1) a single thyroid nodule with a diameter of 5–50 mm, 2) complete clinical and ultrasonic data, and 3) all nodules with definite pathological confirmation. The exclusion criteria were 1) undistinguishable coalescent thyroid lesions and 2) pathology provided ambiguous diagnostic findings for their nodules. Please see **Figure S1**.

### Collection of ultrasound data

Ultrasound images of thyroid glands and the surrounding areas were acquired by ultrasound machine with a linear array probe at Xianyang Central Hospital. The ultrasound images were performed independently by two ultrasonologists, with a senior ultrasonologist making the final decision on controversial patients. The following features of each nodule, including the size, shape, composition, echogenicity, margin, echogenicity, and cervical lymph node status, were carefully measured and recorded. Images of the thyroid are obtained according to ACR accreditation standards. Ultrasound features were divided according to the ACR TIRADS (3), and each feature had a corresponding score. The higher the score, the greater the malignant tendency. In the processing of statistical analysis, the ultrasound characteristics of each nodule were replaced with the corresponding scores in the ACR TIRADS. For example, taller-than-wide will be assigned 3 points, so we wrote the Arabic numeral 3 instead of taller-than-wide in **Table 1**.

**Table 1:** The results of univariate and multivariable logistic regression.

Characteristics	Univariate logistic regression			Multivariable logistic regression		
	OR	CI	p	OR	CI	p
Age	0.97	0.95–0.99	0.002	0.97	0.95–1	0.027
Composition						
1	Ref	Ref	Ref	Ref	Ref	Ref
2	4.8	1–23.03	0.05	NA	NA	NA
Echogenic.Foci						
0	Ref	Ref	Ref	Ref	Ref	Ref
1	1.21	0.54–2.67	0.644	0.75	0.28–1.97	0.557
2	1.48	0.09–24.16	0.781	1.35	0.06–30.07	0.848
3	7.54	3.85–14.76	<0.001	4.12	1.87–9.05	<0.001
Echogenicity						
1	Ref	Ref	Ref	Ref	Ref	Ref
2	9.21	3.95–21.49	<0.001	4.72	1.76–12.68	0.002
3	3.81	0.54–27.08	0.181	3.89	0.47–32.19	0.208
Laterality						
Left	Ref	Ref	Ref	Ref	Ref	Ref
Right	0.91	0.56–1.48	0.7	NA	NA	NA
Middle	3.53	0.95–13.2	0.061	NA	NA	NA
Lymph.Nodes						
No	Ref	Ref	Ref	Ref	Ref	Ref
Yes	6.9	2.8–16.98	<0.001	5.48	1.97–15.27	0.001
Margin						
0	Ref	Ref	Ref	Ref	Ref	Ref
2	6.83	3.31–14.08	<0.001	3.87	1.71–8.77	0.001
3	8.71	3.46–21.91	<0.001	4.61	1.64–12.95	0.004
Shape						
0	Ref	Ref	Ref	Ref	Ref	Ref
3	3.83	2.08–7.06	<0.001	2.86	1.38–5.93	0.005

Composition (1, mixed cystic and solid; 2, solid or almost completely solid). Echogenic.Foci (0, none or large comet-tail artifacts; 1, macrocalcifications; 2, peripheral (rim) calcifications; 3, punctate echogenic foci). Echogenicity (1, hyperechoic or isoechoic; 2, hypoechoic; 3, very hypoechoic). Margin (0, smooth or ill-defined; 2, lobulated or irregular; 3, extra-thyroidal extension). Shape (0, wider-than-tall; 3, taller-than-wide). NA, Not Available.

The benign and malignant pathology of all thyroid nodules in all participants was confirmed by FNA or surgery. All pathological results were examined blindly and separately by two pathologists, with a final decision made by a senior pathologist.

### Analysis strategy

In order to maximize the predictive performance and ultimately reduce overfitting, we used the least absolute shrinkage and selection operator (lasso) regression analysis to screen variables, followed by logistic analysis to identify independent risk factors for malignant nodules.

A total of six ML algorithms were developed in this study, including Decision Tree (DT), Extreme Gradient Boosting (XGBoost), Gradient Boosting Machine (GBM), Naive Bayes Classifier (NBC), Random Forest (RF), and Logistic Regression (LR), to predict malignant thyroid nodules based on the variables with multivariable logistic regression p-value less than 0.05. Models have been validated internally by using 10-fold cross-validation. Subsequently, the area under the receiver operating characteristic curve (AUC) values, accuracy, precision, recall, and F1 score have been calculated to measure and compare the performance of each model.

Because many machine learning algorithms are considered functional black boxes, their internal processes are not well understood. Given this issue, various interpretability methods have been proposed to assess the influence of variables on the predicted results (21, 22). For instance, the relative importance of variables, the Shapley additive explanations (SHAP) method, and the heat map of the correlation of features were employed to further visualize the interpretation of ML-ed models at the feature level. An optimal cutoff value for clinical application was determined by probability density functions (PDFs). Clinical utility curves (CUCs) were plotted to compare the net benefits of different thresholds.

The demographic and clinical characteristics of all included patients were analyzed by t-test and chi-square test *via* SPSS Statistics software (version 26.0, SPSS Inc., Chicago, IL, USA). Continuous and categorical variables are expressed as mean ± SD and frequency in this study. p-Values <0.05 were considered statistically significant with 95% confidence intervals (CIs) applied for all analyses. R software was applied for developing predictive models *via* the “rms” package and establishing a web risk calculator *via* the “shiny” package.

## Results

### Clinical and ultrasound characteristics

Of all 9,999 participants with thyroid nodules, 500 (50%) harbored malignant nodules, while 500 (50%) had benign disease based on the pathological diagnosis. We collected clinical features (age and gender) and recorded image features (thyroid nodule location, size, shape, composition, echogenicity, margin, echogenicity, and cervical lymph node status).

### Selection of features

Eight of 15 variables were screened by lasso analysis into logistic regression analysis, and all statistically significant factors in the univariate logistic regression analysis were included in the multivariate logistic regression analysis. Finally, age, margin, shape, echogenic foci, echogenicity, and lymph nodes were identified as independent predictors of thyroid cancer. There was no significant statistical difference in the nodule location (laterality) and composition in the differentiation of benign and malignant thyroid nodules. The results of the univariate and multivariate analyses are demonstrated in **Table 1**.

### Demographic baseline

A cohort of patients from Xianyang Central Hospital in China was enrolled in this study. Results of the t-test and the chi-square test indicated there was no statistically significant difference between the training and the validation cohorts at a 0.05 significance level.

### Development and validation of ML-ed models

**Figure 1:**
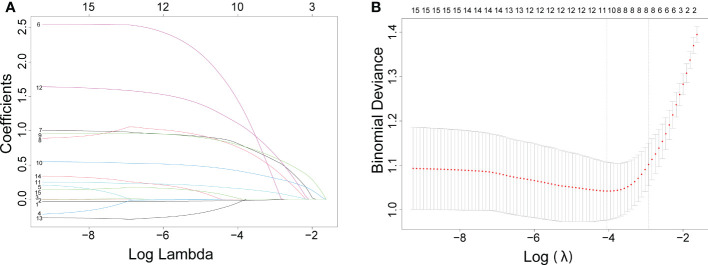
The results of the least absolute shrinkage and selection operator (lasso) regression analysis. The coefficients of all variables are reduced to 0 from instability to stability in **(A)** and obtain the model coefficient of λ value that minimizes the model deviation by cross-validation curve in **(B)**.

**Figure 2:**
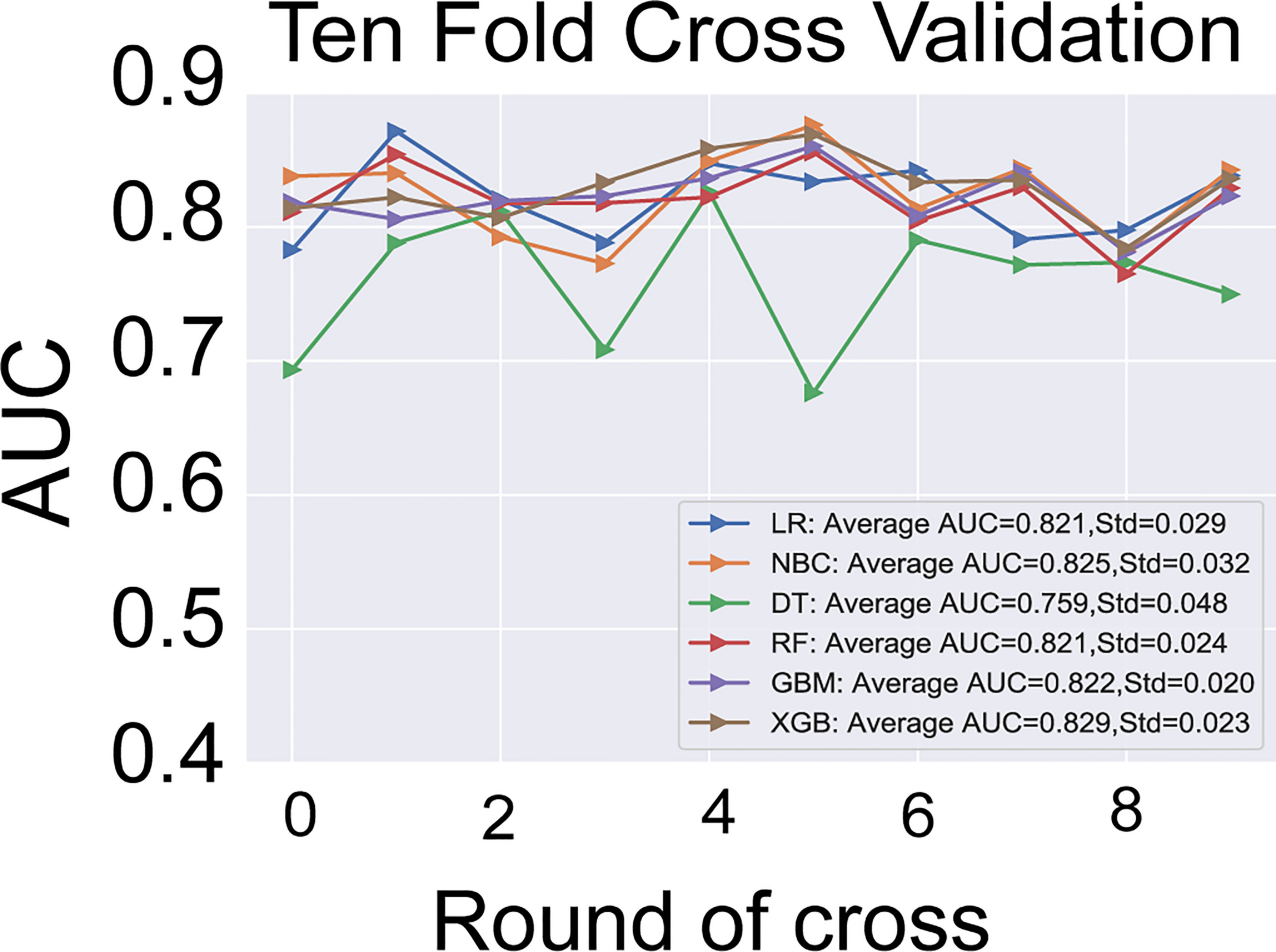
The results of 10-fold cross-validation in the six models of LR, NBC, DT, RF, GBM, and XGBoost. The average AUC of XGBoost model is the highest one. LR, Logistic Regression; NBC, Naive Bayes Classifier; DT, Decision Tree; RF, Random Forest; GBM, Gradient Boosting Machine; XGBoost, Extreme Gradient Boosting; AUC, area under the receiver operating characteristic curve.

The six predictors identified in the differentiation of malignant and benign thyroid nodules were used to construct ML-ed models, including LR, NBC, DT, RF, GBM, and XGBoost, respectively, for predicting malignant thyroid nodules. The predictive performance of the six ML-ed models is shown in **Figure 1**. The whole cohort used 10-fold cross-validation in this study. All models had shown good performance in predicting malignant nodules. Their AUC values of XGBoost, LR, NBC, DT, RF, and GBM were 0.829, 0.821, 0.825, 0.759, 0.821, and 0.822, respectively, in the 10-fold cross-validation. The XGBoost model indicated the best performance than any of the others. Meanwhile, the XGBoost model also achieved the highest accuracy of 0.65 and precision of 0.63, as shown in **Figure 2**. Thus, the XGBoost was identified as our final predictive model in this study.

### Explanation of model

**Figure 3:**
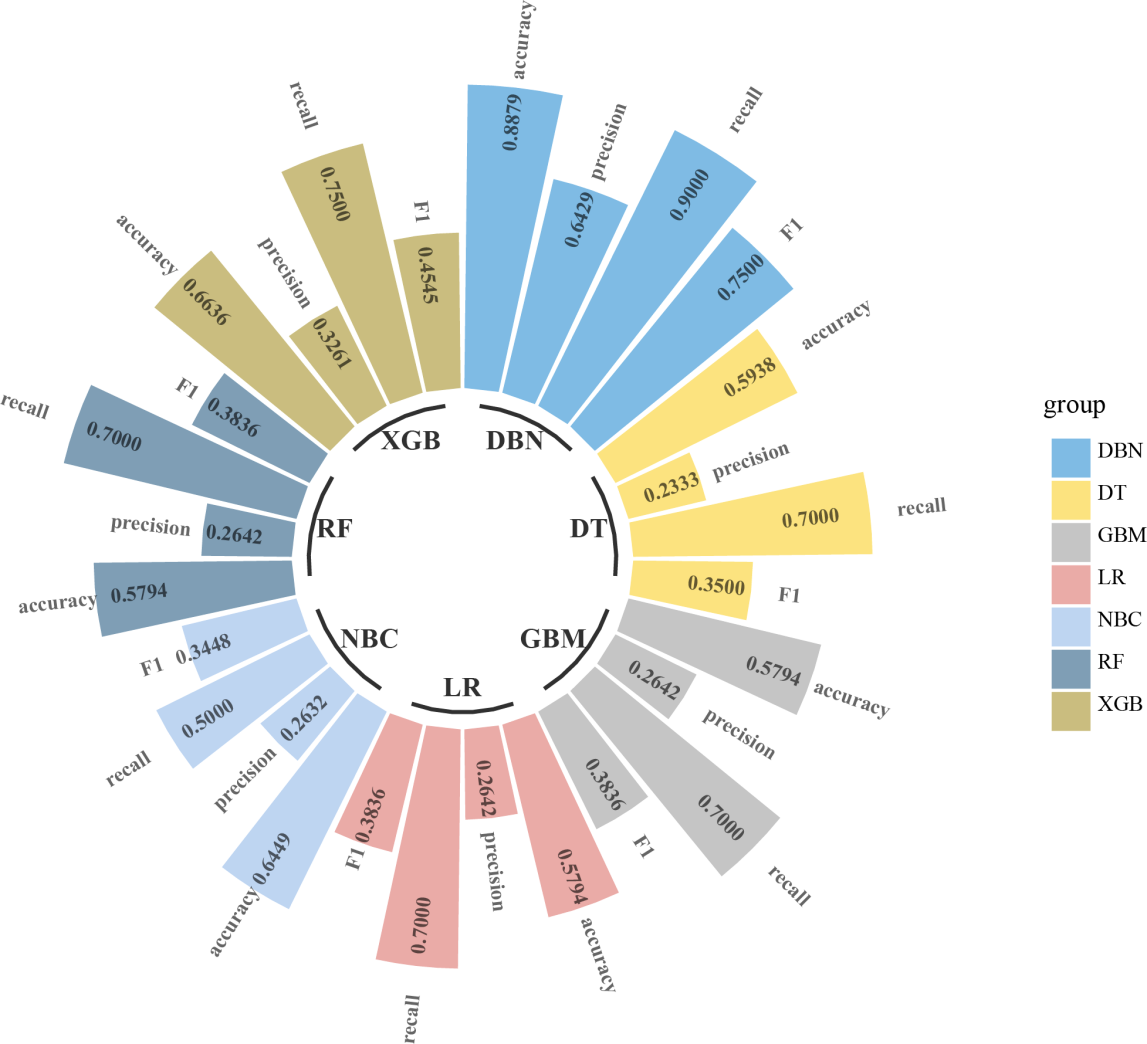
Circular bar plot. The performance of six models has been evaluated by five criteria of AUC, accuracy, precision, recall, and F1. AUC, area under the receiver operating characteristic curve.

To further illustrate the models at the feature level, a SHAP summary diagram was plotted to demonstrate how these features affect the presence of malignant thyroid nodules. The SHAP values for each feature plotted for each sample are shown in **Figure 3**. We concluded that margin, shape, echogenic foci, echogenicity, and lymph nodes exerted negative effects on predicting the risk of malignant thyroid nodules, whereas the risk of malignancy increases with age.

**Figure 4:**
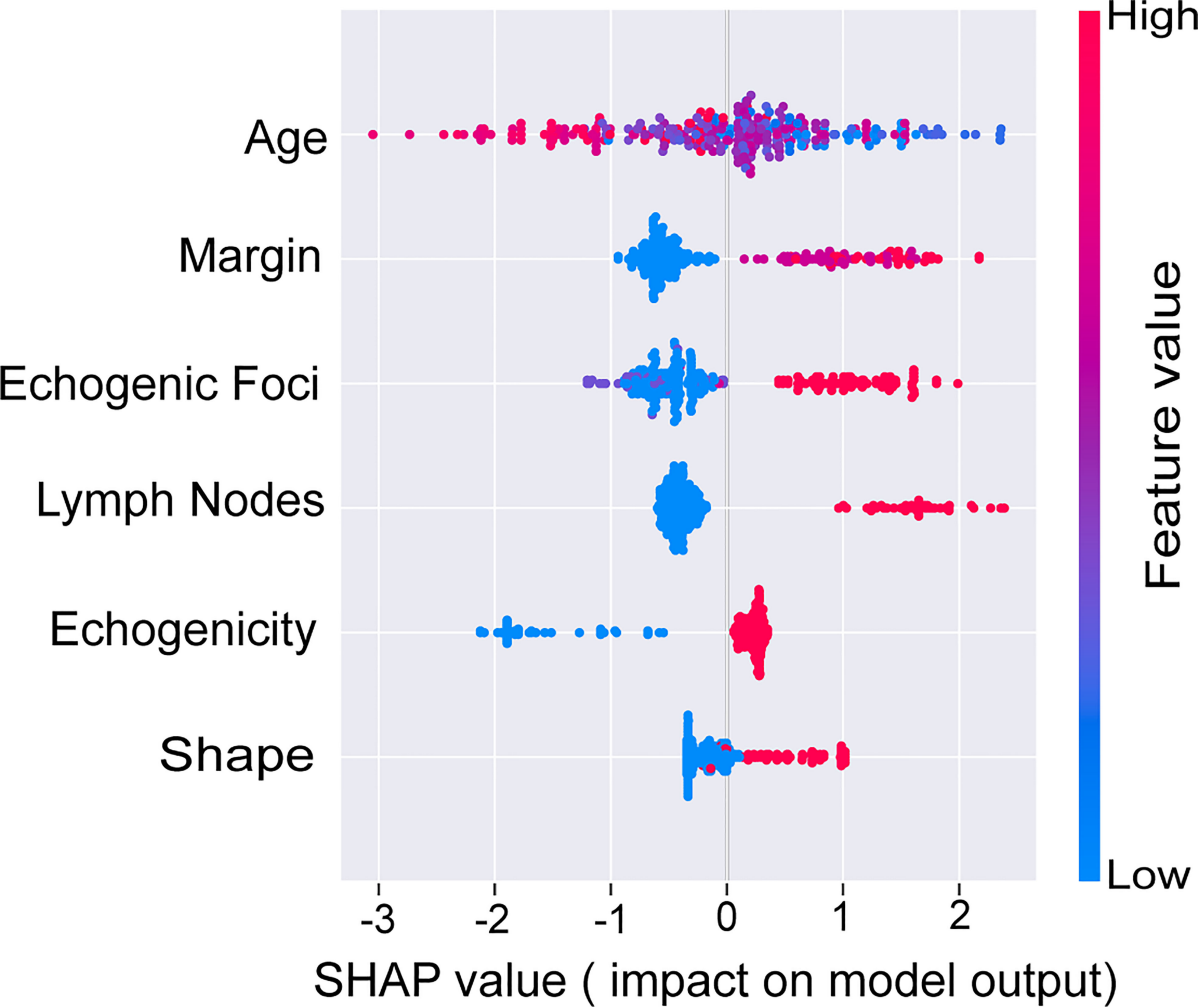
SHAP values of the selected features. The higher the SHAP value of each variable, the more impact and contribution to the model. SHAP, Shapley additive explanations.

**Figure 5:**
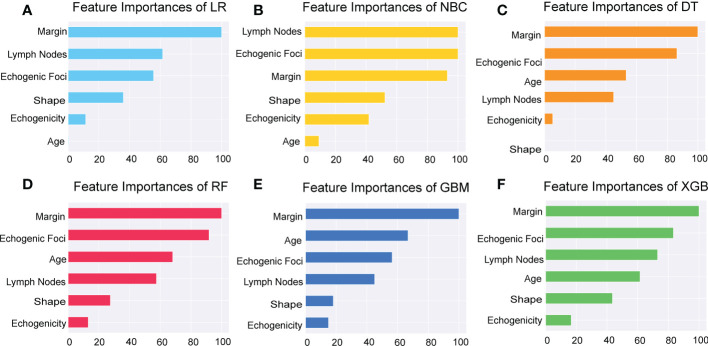
Importance of the selected features. Importance of each feature had been demonstrated and compared in the six models of LR, NBC, DT, RF, GBM, and XGBoost. LR, Logistic Regression; **(A)** NBC, Naive Bayes Classifier; **(B)** DT, Decision Tree; **(C)** RF, Random Forest; **(D)** GBM, Gradient Boosting Machine; **(E)** XGBoost, Extreme Gradient Boosting **(F)**.

Additionally, we ranked the importance of features in **Figure 4** in order to explore the extent to which each independent risk factor contributed to the model. Although there were slight differences in the importance of each variable across models, the margin contributed most to the prediction of malignant nodules in most models. In the XGBoost model, the relative importance of variables decreased in the following order: margin, echogenic foci, lymph nodes, age, shape, and echogenicity. The correlation heat map indicated there was no linear correlation between the variables, and they harbor independent predictive power in clinical practice (**Figure 5**).

### Application of model

**Figure 6:**
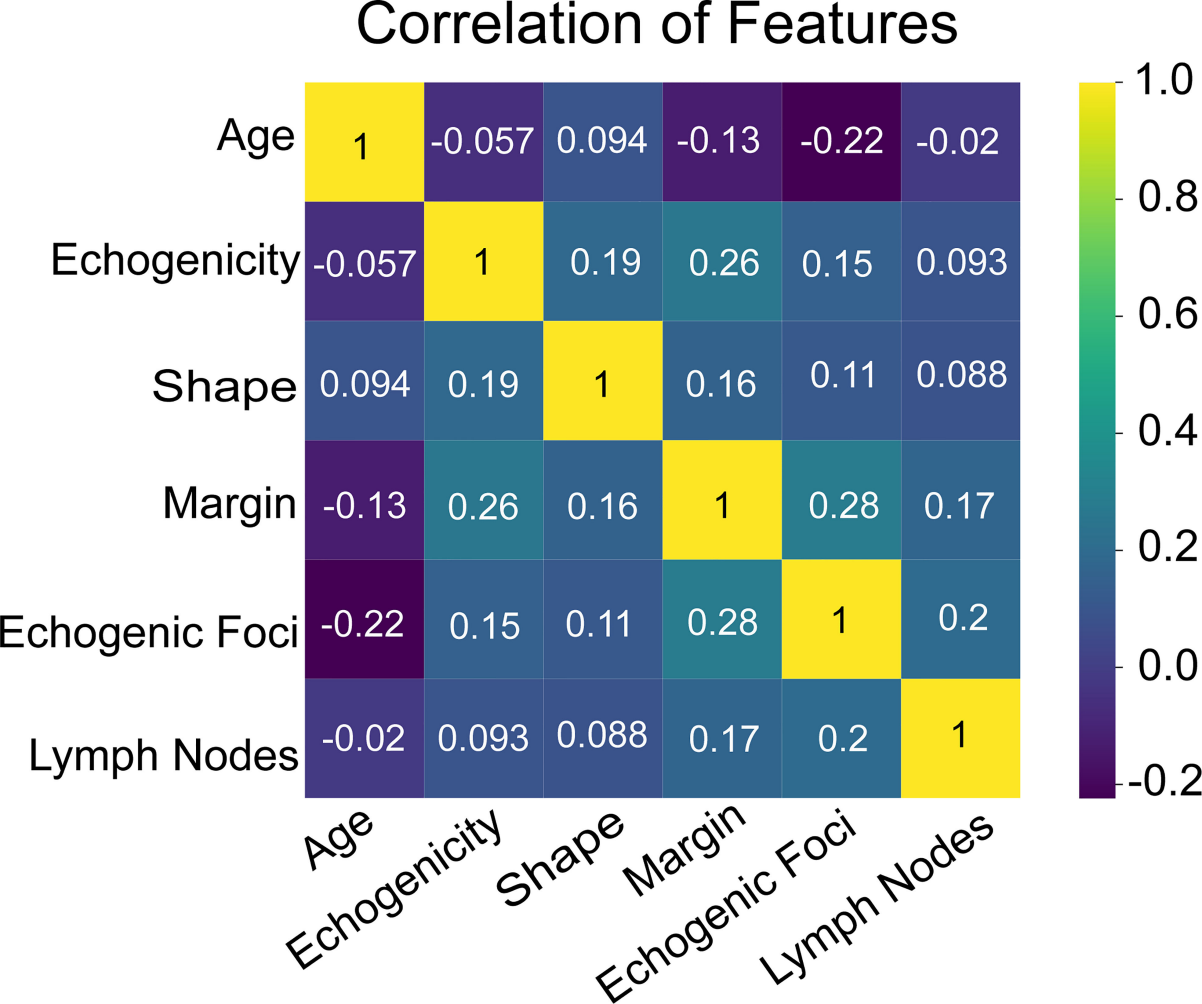
The heat map of correlation of the selected features, including age, echogenicity, shape, margin, echogenic foci, and lymph nodes.

**Figure 7:**
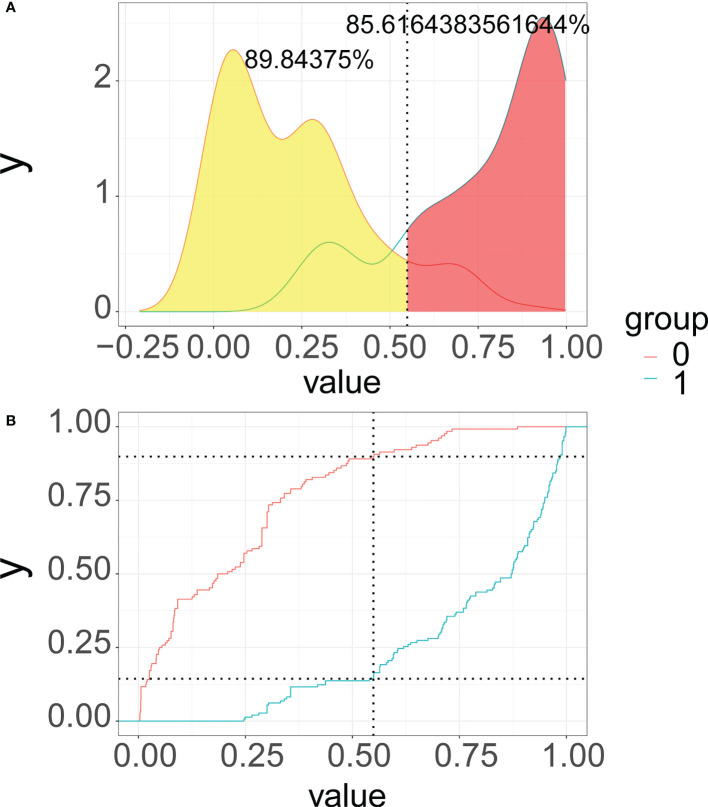
Probability density functions **(A)** and **c**linical utility curves **(B)** of the predictive model (0, benign modules group; 1, malignant nodules group). It is 85.6% (red area under the blue line) of malignant nodules, and the number of biopsy procedures for benign nodules was reduced by 89.8% (yellow area under the red line).

**Figure 8:**
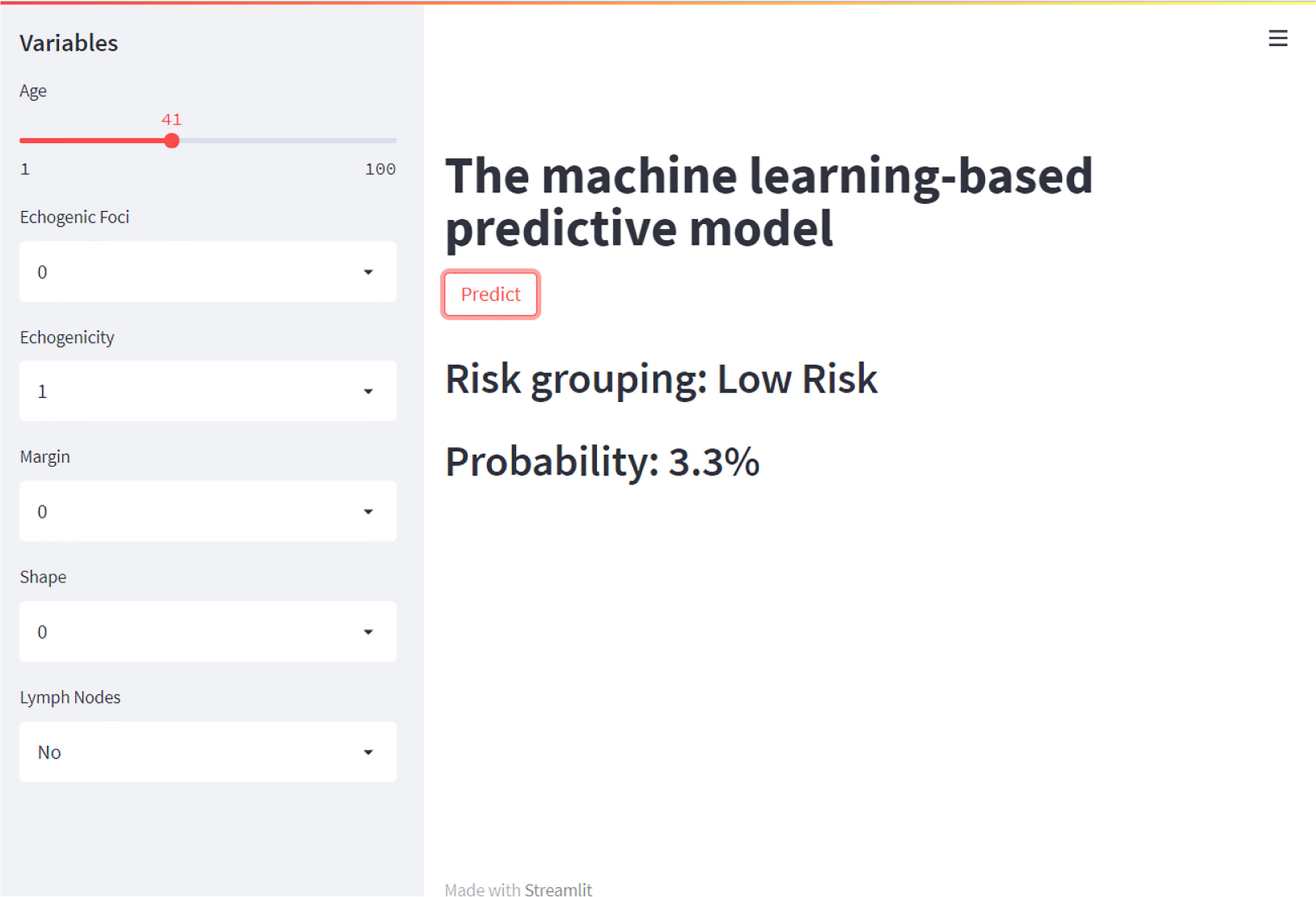
The application of the web risk calculator for patients with malignant nodules.

As illustrated in **Figure 6**, we recommend a threshold probability of 51% as the optimum cutoff value for the probability of malignant nodules. In this situation, we could detect 85.6% (red area under the blue line) of malignant nodules, while the number of biopsy procedures for benign nodules would be reduced by 89.8% (yellow area under the red line) in **Figure 7**. Finally, in order to facilitate the practical application of the model in clinical work, we embedded the best predictive model into a web risk calculator (**Figure 8**) that can easily derive the probability and risk stratification of patients with malignant nodules in real time. **Figure S1** shows the flow chart of our current study.

## Discussion

As a highly prevalent disease, the incidence of thyroid nodules in China is 20%–35% (23), of which 7%–15% are malignant (1). It is a challenge for clinicians to distinguish malignant from benign nodules. Hence, we propose to develop a predictive model based on machine learning for assessing the malignancy risk of thyroid nodules in the Chinese population. To our knowledge, this is the first ML-ed predictive model to predict malignant thyroid nodules by integrating clinical and ultrasound features. Our model was constructed using six variables, including clinical characteristics (age) and ultrasound features (margin, shape, echogenic foci, echogenicity, and lymph nodes). Our findings indicated that the proposed model could detect malignant thyroid nodules accurately and reduce unnecessary biopsies by estimating risk stratification. Finally, through a convenient and practical web application, our model can assist doctors and patients to carry out precise and individualized management of thyroid nodules.

We found that age played an adverse role in the risk of malignancy as a predictive parameter in our model. This finding was consistent with that of Chen et al., where there was no increased risk of malignancy in those aged 28–63 in the same population of a Chinese cohort (24). In addition, a similar finding was revealed in a US cohort of 196 patients with malignant FNA cytology, and the incidence of malignant thyroid nodules in patients under 45 years old was twice that in those over 45 years old (8.1% *vs.* 4.0%, p < 0.001) (25). Similarly, Italian scholars have reported that cytology suspicious or indicative of papillary thyroid cancer is associated with younger age (26). However, our result was contrary to what Belfifiore et al. reported that thyroid cancer is more common in elderly patients (27). Different views on the association between age and the incidence of malignant thyroid nodules deserve further exploration.

Previous findings unveiled that the abnormal cervical lymph nodes may indicate malignant nodule metastasis (28), which is consistent with our study. It is reported that 30%–80% of patients with thyroid cancer have cervical lymph node metastasis (29). Cervical lymph nodes are usually not palpable because of their deep location and small size. Ultrasound has demonstrated its high sensitivity and specificity for the assessment of non-palpable lymph nodes (30). Cervical lymph nodes may enlarge as a result of a benign process, such as reactive hyperplasia due to inflammation in submandibular and upper cervical nodes (29). However, most investigators agree on the sonographic features of metastatic lymph nodes in thyroid cancer, including cystic degeneration, a rounded shape, loss of echogenic hilum, hypoechoic or hyperechoic mass, and calcification (31–33). The cervical lymph node is the first metastatic site of malignant nodules. Thyroid nodules should be highly suspected as malignant when abnormal lymph nodes are observed.

Meanwhile, margin ranked first in the importance of features and contributed the most to our model. We found that lobulated or irregular margins and extensive extrathyroidal extension detected by ultrasound increased the risk of malignancy risk in nodules. A lobulated or irregular margin is defined as a spiculated or jagged edge. Some studies have revealed that an irregular or microlobulated margin suggests malignancy (34, 35). Extensive extrathyroidal extension refers to a frank invasion of adjacent soft tissue or vascular, which is a highly reliable characteristic of malignancy and also has a negative effect on prognosis (36).

Furthermore, another feature we found that increased the risk of malignant nodules was the shape of the nodules. As first observed by Kim et al. and subsequently confirmed in a series of studies (35, 37–40), a lump with a shape taller-than-wide is another useful predictor of malignancy. These results may be associated with the growth pattern. It is found that the growth of benign nodules remains within normal tissue planes, so the shape of benign nodules can be ovoid to round, whereas malignant nodules grow centrifugally through the normal tissue plane (38, 39). In the ACR TIRADS, taller-than-wide was assigned 3 points in the TIRADS, and our results have confirmed the high-risk role of shape in the malignancy of thyroid nodules.

Notwithstanding, echogenic foci, and echogenicity were regarded as predictive variables in both our predictive model and the ACR TIRADS; there were different opinions on assessing thyroid nodules on some features. Our results showed the presence of macrocalcifications and peripheral (rim) calcifications had no statistical difference between malignant and benign nodules. However, macrocalcifications and peripheral (rim) calcifications could be assigned 1 and 2 points in the TIRADS, respectively. Macrocalcifications refer to coarse echogenic foci accompanied by acoustic shadowing. Evidence in published data describing their correlation with an increased malignancy risk is weak (41); additionally, the relationship between macrocalcifications and nodules lacking other malignant characteristics is also mixed (42, 43). Peripheral (rim) calcifications lie along all or part of the nodule’s margin. Compared with macrocalcifications, they are more strongly correlated with malignancy (41), but several studies suggested that their correlation with malignancy is variable (43).

The statistical results of logistic regression showed that patients with punctate echogenic foci had a higher tendency to develop malignant nodules. Punctate echogenic foci are smaller in size and less shadowed than macrocalcifications and may correspond to the psammomatous calcifications associated with papillary cancers in the solid components of thyroid nodules. Histologically, punctate echogenic foci are smaller and less shadowed than macrocalcifications, which are considered highly positive associations with malignancy, especially in combination with other suspicious features (3, 5).

Echogenicity refers to a nodule’s reflectivity relative to adjacent tissue. Except for the thyroid parenchyma, which is usually used as reference tissue, the neck strap muscles with very low echogenicity are also used as the basis for comparison. Previously, several studies investigated that a higher degree of hypoechogenicity was highly suggestive of malignancy, with a specificity of 92%–94% (37, 44). Interestingly, a higher degree of hypoechogenicity harbors no statistical significance for predicting malignant nodules based on our results of multivariable logistic regression. These results need more evidence to verify.

Another important finding of our paper was composition, which was not an independent predictor of malignant nodules. Thyroid nodules that are cystic or almost completely cystic have no score in the ACR TIRADS because they are highly correlated with benign cytology, and only 13%–26% of thyroid cancers harbor a cystic component (29, 44–46). Spongiform, composed predominantly (>50%) of small cystic spaces, is considered a sign of benignity with high specificity (44). In our study, we found that no patient had cystic or almost completely cystic or spongiform ultrasound features. Additionally, according to ACR TIRADS, mixed cystic and solid, and solid or almost completely solid are the risk factors for malignant nodules, with scores of 1 and 2, respectively (3). Solid nodules with an eccentric configuration and acute angle are suspected to be malignant (47), whereas these conclusions were not observed in our study.

The differences between our study and the risk stratification system also illustrate the inadequacy of the classification system to evaluate thyroid nodules, such as interobserver variation/a subjective assessment of the nodules (8, 48). Therefore, it is necessary to add clinical data to further improve the accuracy and objectivity of the predictive model. As Chen et al. described in their literature, the predictive power of the new model was superior to that of ACR TIRADS when age is included. Furthermore, our study cohort enrolled retrospectively Chinese patients from a single medical center; these differences therefore may be due to demographic differences and healthcare disparities between patients in the USA and China.

Therefore, these differences may be due to the mismatch between the classification system and the current medical situation in China. It is more rational to apply a risk stratification system according to the population. Accordingly, Zhou et al. formulated Chinese guidelines for ultrasound malignancy risk stratification of thyroid nodules (C-TIRADS) that are specific to China’s national and medical conditions (23).

We offered clinicians and patients an online web application for estimating the risk of a malignant thyroid nodule using the XGBoost model, which combined six variables including age, lymph nodes, margin, shape, echogenic foci, and echogenicity. By inputting the corresponding personalized parameters of patients, visitors can quickly obtain the corresponding malignancy risk. The link is as follows: https://share.streamlit.io/liuwencai6/thyroid_final/main/thyroid_final.py.

Depending on the cutoff value in the PDF and CUC, we recommended 51% as the threshold probability of the next management strategy and risk stratification. In that case, about 85.6% of patients with malignant thyroid nodules can be detected, FNA was recommended, and careful follow-up and possibly early surgery should be considered. Moreover, we could also save approximately 89.8% of unnecessary biopsy procedures in low-risk populations (malignant risk ≤51%). This result is consistent with the main goal of all currently available sonographic risk stratification systems, that is, to eliminate unnecessary thyroid biopsies without endangering the diagnosis of clinically malignant nodules (15). We believe that the incorporation of our predictive model into clinical practice will improve the diagnostic accuracy of malignant thyroid nodules and minimize the number of unnecessary FNA in low-risk patients with thyroid nodules. Compared with existing models (49, 50), the performances are good but various due to differences in population and dataset.

There are several limitations of this existing study. First, the retrospective nature of this study may have resulted in potential bias. Second, the ultrasound features were read and provided by sonographers rather than captured directly from the ultrasonic images, which may cause bias in data quality due to extraction and interpretation. We strongly recommend that the machine learning model be used to extract the features from ultrasonic images directly and from several types of machines in future studies. In addition, all patients and ultrasound assessments are derived from a single medical center, which may restrict the accuracy; large-scale multicenter cohorts and external validation would be more forceful. Finally, we classified the features of nodules by ACR TIRADS, rather than the Chinese-TIRADS proposed by the Chinese professional society, to evaluate ultrasound parameters (23). We will expand our cohort and dataset in a further study to optimize our model and algorithm in the future.

## Conclusion

In conclusion, our study yielded a machine learning-based model combining age with ultrasound parameters, including shape, margin, echogenic foci, echogenicity, and lymph nodes, to predict the presence of malignant thyroid nodules. Our model showed good performance and was embodied in a web risk calculator to estimate the risk of malignant thyroid nodules.

## Data Availability

The raw data supporting the conclusions of this article will be made available by the authors, without undue reservation.

## Supplementary material

SUPPLEMENTARY FIGURE 1 Flow Chart.
